# Supplementary material for: Severe COVID-19 Shares a Common Neutrophil Activation Signature with Other Acute Inflammatory States
Source: Cells. 2022 Mar 1;11(5):847. doi: 10.3390/cells11050847 (PMC8909161; doi:10.3390/cells11050847)
Supplement: Supplementary file 1 [file cells-11-00847-s001.zip › Key resource table Methods LF Schimke 2022.pdf]

KEY RESOURCES TABLE

REAGENT or RESOURCE	SOURCE	IDENTIFIER
**Deposited data**		
mircoarray data of HLH patients and healthy controls	Sumegi et al. 2011	GEO: GSE26050
bulk-RNA seq data of COVID-19 patients and controls	Arunachalam et al. 2020	GEO: GSE152418
bulk-RNA seq data of COVID-19 patients and controls	Liebermann et al. 2020	GEO: GSE152075
bulk-RNA seq data of COVID-19 patients and patients with other respiratory diseases	Mick et al. 2020	GEO: GSE156063
bulk-RNA seq data of COVID-19 patients and controls	Ng et al. 2021	GEO: GSE163151
bulk-RNA seq data of COVID-19 patients and controls	Thair et al. 2021	GEO: GSE152641
bulk-RNA seq data of COVID-19 patients and patients with other respiratory symptoms	Overmyer et al. 2020	GEO: GSE157103
scRNA seq data of COVID-19 patients and controls	Schulte-Schrepping et al. 2020	EGA: EGAS00001004571
bulk-RNA seq data of COVID-19 patients, healthy controls, and patients with Influenza, bacterial pneumonia, and seasonal Coronavirus other than SARS-CoV-2	McClain et al.2021	GSE161731
bulk-RNA seq data of patients with multisystem inflammatory syndrome in children (MIS-C) and healthy controls	Beckmann et al. 2021	GSE178388
microarray data of patients with Kawasaky disease (KD) and healthy controls	Wright et al. 2018	GSE73461
**Software and algorithms**		
NetworkAnalyst 3.0	Zhou, G. et al. 2019	https://www.networkanalyst.ca/
Limma voom pipeline	Law, C. W. et al. 2014	https://doi.org/10.1186/gb-2014-15-2-r29
jvenn	Bardou, P. et al. 2014	http://jvenn.toulouse.inra.fr/app/example.html
Circos online tool	Krzywinski, M. et al. 2009	http://mkweb.bcgsc.ca/tableviewer/
Seurat V.4	Hao, Y. et al. 2020	https://doi.org/10.1016/j.cell.2021.04.048https://satijalab.org/seurat/
NAViGaTOR 3.0.14	Brown, K. R. et al. 2009	doi:10.1093/bioinformatics/btp595
Integrated Interactions Database, IID version 2020-05	Kotlyar M. et al. 2018	http://ophid.utoronto.ca/iid
ClusterProfiler	Yu, G et al. 2012	doi:10.1089/omi.2011.0118.
Enrichr	Chen, E. Y. et al. 2013; Kuleshov, M. V. et al.2016	https://maayanlab.cloud/Enrichr/
Morpheus	Starruß, J. et al. 2014	https://software.broadinstitute.org/morpheus/
R version 4.0.5	R Core Team (2020)	https://www.r-project.org/

R studio Version 1.4.1106	RStudio Team (2020)	http://www.rstudio.com/.
circlize R package	Zuguang Gu et al. 2020	https://github.com/jokergoo/circlize
ggpubr R package	Alboukadel Kassambara, 2020	https://rpkgs.datanovia.com/ggpubr/
lemon R package	Stefan McKinnon Edwards et al. 2020	https://github.com/stefanedwards/lemon
ggplot2 R package	Wickham H, 2016	https://ggplot2.tidyverse.org
factoextra R package for PCA analysis	Alboukadel Kassambara and Fabian Mundt, 2017	https://rpkgs.datanovia.com/factoextra/index.html
Canonical Correlation Analysis (CCA) R package	Jendoubi, T. & Strimmer, K., 2019	https://cran.r-project.org/web/packages/whitening/index.html http://www.strimmerlab.org/software/whitening/
corrgram R package	Kevin Wright, 2021	https://kwstat.github.io/corrgram/
psych R package	William Revelle, 2021	https://personality-project.org/r/psych/
inlmisc R package	Jason C Fisher, 2021	https://github.com/USGS-R/inlmisc
tydiverse R package	Hadley Wickham, 2021	https://github.com/tidyverse/tidyverse/
viridis R package	Simon Garnier et al. 2021	https://sjmgarnier.github.io/viridis/
randomForest (version 4.6.14)	Andy Liaw and Matthew Wiener 2002	https://cran.r-project.org/doc/Rnews/Rnews_2002-3.pdf
Fisher´s method	Fisher, R. A., 1932	Fisher, R. A. Statistical Methods for Research Workers 4th edn. (Oliver and Boyd, 1932).
nonparametric MANOVA: nparcomp R package	Konietschke, F. et al., 2015	doi:10.18637/jss.v064.i09
nonparametric Inference for Multivariate Data: npmv R package	Burchett, W. et al., 2017	doi:10.18637/jss.v076.i04
